# Chrysophanol Exerts Anti-inflammatory Activity by Targeting Histone Deacetylase 3 Through the High Mobility Group Protein 1-Nuclear Transcription Factor-Kappa B Signaling Pathway *in vivo* and *in vitro*

**DOI:** 10.3389/fbioe.2020.623866

**Published:** 2021-01-25

**Authors:** Quan Wen, Ngaikeung Lau, Huandi Weng, Peng Ye, Shaohui Du, Chun Li, Jianping Lv, Hui Li

**Affiliations:** ^1^Guangdong-HongKong-Macau Institute of CNS Regeneration, Jinan University, Guangzhou, China; ^2^School of Basic Medical Sciences, Guangzhou University of Chinese Medicine, Guangzhou, China; ^3^Guangdong Provincial Hospital of Chinese Medicine, Guangzhou, China; ^4^Shenzhen Affiliated Hospital, Guangzhou University of Chinese Medicine, Guangzhou, China; ^5^School of Nursing Sciences, Guangzhou University of Chinese Medicine, Guangzhou, China; ^6^Department of Neurosurgery, Guangzhou First People’s Hospital, School of Medicine, South China University of Technology, Guangzhou, China

**Keywords:** chrysophanol, sepsis, HMGB1, HDAC3, NF-κB

## Abstract

Chrysophanol (Chr) is the main monomer isolated from *Rheum rhabarbarum*. This study aimed to identify the potential *in vitro* and *in vivo* cytoprotective effects of Chr on lipopolysaccharide (LPS)-triggered acute lung injury (ALI). We used an ALI-murine model and constructed an inflammatory macrophage *in vitro* cell model to determine the cellular mechanisms involved in Chr-mediated activity. To observe the vital role of histone deacetylase 3 (HDAC3) in abolishing inflammation action, HDAC3 was downregulated using small interfering RNA. Analysis of the expression of nuclear transcription factor-kappa B p65 (NF-κB p65) and molecules of its downstream signaling pathway were assessed *in vitro* and in lung tissue samples using the mouse model. Concentrations of tumor necrosis factor-α, interleukin-1β, high mobility group protein 1 (HMGB1), and interleukin-16 in supernatants and the bronchoalveolar lavage fluid were measured using enzyme-linked immunosorbent assay. A reporter gene assay measured HMGB1 activity, and NF-κB p65 and HMGB1 intracellular localization was determined by immunofluorescence detection on histological lung samples from Chr-treated mice. The protein interactions between HMGB1, HDAC3, and NF-κB p65 were tested by co-immunoprecipitation. Chr treatment relieved LPS-induced lung lesions. Chr also enhanced superoxide dismutase levels in ALI mice. Chr reduced the LPS-induced protein expression of NF-κB and its related pathway molecules in both *in vivo* and *in vitro* models. Moreover, Chr downregulated LPS-enhanced HMGB1 expression, acetylation, and nuclear nucleocytoplasmic translocation. However, HDAC3 knockdown substantially reduced Chr-mediated HDAC3/NF-κB expression.

Furthermore, Chr enhanced HMGB1/HDAC3/NF-κB p65 complex interaction, whereas HDAC3 knockdown reduced Chr-mediated HMGB1/HDAC3/NF-κB p65 formation. This study showed that the protective effects induced by Chr were associated with the regulation of the HMGB1/NF-κB pathway via HDAC3.

## Graphical Abstract

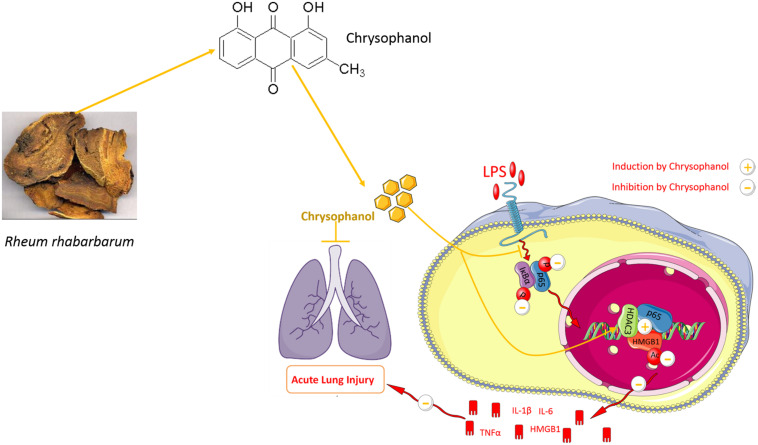

## Introduction

Sepsis, a generalized inflammatory state induced by lipopolysaccharide (LPS) infection, usually leads to acute lung injury (ALI). Sepsis can induce multi-organ dysfunction syndrome or septic shock (Drewry et al., 2017). Septic ALI is induced by a lung inflammatory response syndrome, which leads to high mortality rates, high patient management costs, and accelerated morbidity (Ishii, 2011). LPS is the main component of the outer membrane of Gram-negative bacteria and induces lung leukocyte activation and promotes the secretion of inflammatory cytokines. Effective treatment for sepsis shock for ALI remains limited, and it is necessary to find novel agents.

As a late pro-inflammatory factor, the extracellular high mobility group box 1 (HMGB1) protein triggers responses that cause damage to tissues leading to the activation of the inflammatory cascade in several conditions, including lung injury and septic shock (Czura et al., 2004; El Gazzar, 2007). LPS diffuses into the lung *via* blood circulation, activates macrophages, and promotes HMGB1 release. Extracellular HMGB1 recognizes and binds to its receptors, for example, Toll-like receptor 4, and may activate NF-κB by its translocation to the nucleus where it triggers the transcription and release of inflammatory mediators, including tumor necrosis factor-α (TNF-α), interleukin (IL)-1β, and IL-6 (Valdes-Ferrer et al., 2015). Therefore, HMGB1/NF-κB activation and nucleocytoplasmic transport might show promise as a crucial pathway in the development of ALI.

A crucial epigenetic factor present in macrophages is a histone deacetylase (HDAC). Its activation is triggered by transient inflammatory stimuli and results in the increased synthesis and release of pro-inflammatory cytokines in numerous diseases, including septic shock and ALI. In the latter, attenuation of HDAC activity has been reported to decrease the expression of inflammatory cytokines (Leoni et al., 2002; Pooladanda et al., 2019). Moreover, histone deacetylase 3 (HDAC3) has been reported to promote hyperacetylation and translocation of HMGB1, suggesting a pivotal role for HDACs in regulating the HMGB1 translocation (Zou and Crews, 2014; Banerjee et al., 2015; Pooladanda et al., 2019). Conversely, inhibition of HDAC3 decreased TNF-α levels and was accompanied by a co-dependent increase in acetylated p65, a subunit of NF-κB, which has been reported to play a vital role in abolishing IκBα-mediated NF-κB transcriptional activity (Kiernan et al., 2003; Zhu et al., 2010).

Rhubarb (*Rheum rhabarbarum*) is a well-known traditional medicinal herb that has been widely used in clinical and pharmacological studies. Chrysophanol (Chr), a bioactive compound isolated from Rhubarb, has been reported to protect the human body from LPS-induced toxicity (Kim et al., 2010; Menghini et al., 2016). In the treatment of lung diseases, Chr can reverse lung injury via its anti-inflammatory and immunosuppressive effects (Qian et al., 2011; Lian et al., 2017). Our previous studies have reported that Chr inhibited the LPS-triggered release of pro-inflammatory factors, downregulating NF-κB gene expression and activation *via* regulation of the PPARγ pathway (Wen et al., 2018); however, the detailed molecular mechanisms involved in Chr-mediated effects on the NF-κB pathway are still elusive.

Hence, we propose that Chr attenuates macrophage activation, reduces levels of intrapulmonary inflammation-associated cytokines, and improves LPS-induced sepsis shock through the HMGB1/NF-κB axis by regulating HDAC3.

## Materials and Methods

### Reagents

Chr (purity ≥ 99%) was obtained from the National Institute for the Control of Pharmaceutical and Biological Products (Beijing, China). LPS (055:B5), the HMGB1 antagonist sodium butyrate (SB), and the HDAC3 antagonist RGFP966 were obtained from MedChemExpress (New Jersey, United States). Dexamethasone (DEX) was purchased from Shanghai Winherb Medical Technology Co., Ltd. (Shanghai, China). TNF-α, IL-6, IL-1β, and HMGB1 enzyme-linked immunosorbent assay (ELISA) kits were purchased from Multi Sciences Biotech Co. Ltd. (Hangzhou, China). Malondialdehyde (MDA), myeloperoxidase (MPO), and superoxide dismutase (SOD) kits were purchased from the Jiancheng Bioengineering Institute (Nanjing, China). Dual-Luciferase^®^ reporter assay kits were purchased from Promega (Madison, WI, United States). Antibodies against HMGB1 (#6893), HDAC3 (#3949), TNF-α (#11948), IL-1β (#12703), IL-6 (#12912), inhibitor of nuclear factor kappa B (IκBα) (#4814), acetylated-lysine (#9441), NF-κB p65 (#8242), β-actin (#4970), lamin B (#13435), phospho-p65 (#3031), and phospho-IκBα (#2859) were purchased from Cell Signaling Technology (Danvers, MA, United States). All reagents used for cell culture were obtained from Gibco (Grand Island, NY, United States).

### Experimental Animals and Protocols

Male BALB/c mice (18–22 g) were supplied and housed in the laboratory animal services center (Guangzhou University of Chinese Medicine, Guangzhou, China). The animals were housed in specific pathogen-free surroundings, provided with food and sterilized water *ad libitum*, and exposed to 12 h light/dark cycles and appropriate temperature and humidity. All experimental methods were followed in accordance with the Institutional and National Institutes of Health guidelines for humane animal experimentation. Animal handling followed the dictates of the National Animal Welfare Law of China.

#### Measurement of Blood Pressure and Mortality

Sodium pentobarbital (30 mg/kg) was used to anesthetize mice by intraperitoneal injection. To monitor blood pressure and drug administration, the neck skin was dissected to expose and cannulate the right carotid artery and connected to a blood pressure transducer (BL-420 Apparatus). The mice were grouped into the control group (isotonic saline, *n* = 12) and the LPS-treated group (15 mg/kg, *n* = 72). After LPS treatment, mice went into shock (30 min later, blood pressure reduced by ∼30%); we divided the 72 shock-induced mice into six groups: six groups received increasing does of Chr (7.5, 15, and 30 mg/kg) (Kim et al., 2010; Lian et al., 2017), the DEX treatment group (2 mg/kg) and the LPS group (LPS only). Every 30 min, the blood pressure [mean arterial pressure (MAP)] was recorded for 5 h. The survival rate was tested at the 24 h time point to assess the efficacy of Chr treatment.

#### Tissue Extraction

Mice (*n* = 12 per group) were killed after 24 h, the right lung tissues were rapidly collected under aseptic conditions, and after washing, approximately 50% of the total lung tissue was transferred to −80°C for subsequent quantitative real-time polymerase chain reaction or Western blotting (WB), and another portion of the tissue was fixed in 10% formalin for histological studies.

#### Histological Analysis

The fixed left lungs were rendered transparent for 20 h, embedded in paraffin wax, and sliced into 4 μm sections. After staining, the morphological structure and pathological index of the lung tissues were determined under a light microscope. The lung inflammation was scored by its histological severity as follows: grade 0: no inflammatory cells, grade (1) few cells, grade (2) a ring of cells surrounding the vessels with infiltration 1 cell layer deep, (3) a ring of cells 2–4 cell layers deep; and (4) a ring of cells with more than 4 cell layers deep.

#### Evaluation of Lung Wet-to-Dry Ratio

After blunt dissection, the right lung trachea and esophagus were exposed from the right middle lobe, and the level of pulmonary edema was determined. Next, samples were placed in an oven at 60°C for 48 h and allowed to dry by eliminating moisture, then wet-to-dry (W/D) ratios were calculated.

#### Collection of Bronchoalveolar Lavage Fluid

After treatment with Chr, lung tissue samples were collected and infused three times in phosphate-buffered saline. Bronchoalveolar lavage fluid (BALF) fluid was collected, centrifuged, and then stored at −80°C for subsequent analyses.

### Biochemical Tests

The activities of MPO, SOD, and MDA in the BALF samples from the mouse model were detected following the manufacturer’s instructions using a commercial detection product (Jiancheng Bioengineering).

### Cell Culture and Treatment

RAW264.7 cells (China Center for Type Culture Collection, Shanghai, China) were cultured in Dulbecco’s modified Eagle’s medium supplemented with 10% (v/v) fetal bovine serum, 100 U/ml penicillin, and 100 mg/ml streptomycin and incubated at 37°C in 5% carbon dioxide. There were six groups: control, LPS (0.2 μg/ml), LPS-SB (10 mM SB plus 0.2 μg/ml LPS), and LPS-Chr groups treated with increasing doses of Chr (5, 10, and 5 μM plus 0.2 μg/ml LPS) (Wen et al., 2018). We stimulated cells by LPS for 0.5 h before Chr was added.

### MTT Assay

The MTT assay detected cell activity. First, RAW264.7 cells (5 × 10^3^ cells/ml) were seeded in a 96-well plate overnight. The cells were treated with increasing concentrations of Chr (5, 10, 15, and 20 μM) for an additional 24 h. Next, MTT was added for the final 4 h of culture at 37°C away from light, and the absorbance values were detected at 480 nm.

### Transient Transfection and Luciferase Reporter Assays

RAW264.7 cells were seeded in 24-well plates for 12 h. HMGB1 promoter–reporter plasmids and corresponding negative control (NC) vectors were transiently transfected into cells using Lipofectamine 2000 (Invitrogen, United States). Transfected cells were treated with LPS and/or Chr for another 24 h. Luminescence was determined by the Dual-Luciferase Reporter Assay System (Promega). The ratio of Firefly luciferase/Renilla luciferase was used to normalize the luminescence intensity.

### Total RNA Extraction and Quantitative Real-Time Polymerase Chain Reaction

RAW264.7 cells or lung tissue samples were lysed by TRIzol reagent (Invitrogen). For qRT-PCR, the reaction steps were performed according to the protocol indicated by the manufacturer (Fermentas, United States). Thermal cycling conditions were 9 s at 95°C, 5 s at 95°C, followed by 40 cycles at 95°C for 13 s, 60°C for 1 min, and 95°C for 17 s in a StepOnePlus thermocycler (Applied Biosystems). The primer sequences are listed in Table 1.

**TABLE 1:** Primers for real time-PCR.

Gene	Forward primer	Reverse primer	Species
GAPDH	CGTGTTCCTACCCCCAATGT	TGTCATCATACTTGGCAGGTTTCT	Mouse
HMGB1	GTTCAAGGACCCCAATGCAC	TGGATAAGCCAGGATGCTCG	Mouse
HDAC3	CAGAACTCACGCCAGTATCTGG	TCTGCCGGGACATCATGAAT	Mouse
NF-kB	CGCAAGCCCTTCAGTGACATC	GGTACTGGCTGTCAGGGTGGTT	Mouse
IL-1β	TCGCAGCAGCACATCAACAAGAG	TGCTCATGTCCTCATCCTGGAAGG	Mouse
TNF-α	ATGTCTCAGCCTCTTCTCATTC	GCTTGTCACTCGAATTTTGAGA	Mouse

### Immunofluorescence

After fixation in 3% paraformaldehyde for 40 min, cells were permeabilized by 0.1% Triton X-100 for 30 min and then incubated using the following antibodies overnight at 4°C: rabbit anti-HMGB1 Abs (1:500, CST) and rabbit anti-NF-κB p65 antibody (1:500, CST). Next, cells were exposed to fluorescein isothiocyanate-conjugated secondary antibody (1:1,000, Abcam) for another 1 h, followed by observation by confocal microscopy (Zeiss LSM 710 Meta; Carl Zeiss).

### Cytokine Assay by Enzyme-Linked Immunosorbent Assay

ELISA kits were used to measure the levels of inflammatory factors (TNF-α, IL-6, IL-1β, and HMGB1) in supernatant or tissues in accordance with the manufacturer’s protocols. The emission absorbance of 480 nm was detected using a Thermomax microplate reader.

### Preparation of Protein Extracts and Western Blotting

Briefly, cells were washed, lysed, and centrifuged. Next, cytoplasmic protein extracts were obtained, and the pellets containing nuclei were resuspended, placed on ice for 20 min, and the nuclear debris was centrifuged at 15,000×*g* for 15 min. Cells were lysed in mammalian protein extraction reagent (Thermo Fisher Scientific, Pierce). Total proteins and nuclear and cytoplasmic fractions were collected. The protein concentrations were analyzed using the bicinchoninic acid protein assay kit (Thermo Fisher Scientific, Pierce) and were stored at −80°C until use. The lung tissues were quickly lysed using ice–cold radioimmunoprecipitation assay lysis buffer.

The concentration of total protein of lung tissue lysates was determined for WB. After electrophoresis, proteins were transferred into polyvinylidene fluoride membranes (Roche Ltd, Basel, Switzerland). Membranes were incubated with 5% bovine serum albumin at 37°C for 1 h to block membranes, which were subsequently exposed to the following primary antibodies at 4°C overnight: anti-acetylated lysine (1:1,000), anti-β-actin (1:1,000), anti-glyceraldehyde 3-phosphate dehydrogenase (1:1,000), anti-NF-kB p65 (1:1,000), anti-IkBa (1:1,000), anti-TNF-a (1:1,000), anti-IL-1β (1:1,000), anti-lamin B (1:1,000), anti-HMGB1 (1:1,000), and anti-HDAC3 (1:1,000). Next, an anti-rabbit secondary antibody (1:2,000) was used to incubate the membranes at 37°C for 1 h. The Bio-Rad imaging system imaged the immunoreactive bands.

### Small Interfering RNA Transfection

HDAC3 small interfering RNA (siRNA) and the non-specific NC were constructed by GenePharma (Shanghai, China). The transient siRNA transfection was performed by the protocol indicated by the manufacturer. Cells were transfected for 24 h and then treated with 0.2 μg/ml LPS for 30 min; after that, cells were treated with increasing concentrations of Chr (5, 10, 15, and 20 μM) for 24 h. At the end of the treatment period, we collected cells for WB and immunofluorescence analysis.

### Co-immunoprecipitation

Protein immunoprecipitation (IP) was carried out with antibodies against HMGB1, HDAC3, and NF-κB (CST). Immunoglobulin G was used as a parallel-group. First, samples were incubated with immunoglobulin G reagent to be precleared. Samples were then treated with anti-HMGB1 (1:500), HDAC3 (1:500), and NF-κB (1:500) antibodies; 24 h later, lysates were incubated with protein A/G-agarose for another 2 h. Finally, samples were washed three times in phosphate-buffered saline and subjected to WB analysis.

### Statistical Analysis

GraphPad Prism version 6.0 (GraphPad Software, La Jolla, CA, United States) was used to evaluate the data. Multiple comparisons between the groups were performed using one-way analysis of variance, followed by variance with Tukey’s test. A value of *P* < 0.05 was considered statistically significant; data are expressed as the means ± *SD*.

## Results

### Chrysophanol Treatment Improved the Pathological Changes in Lung Tissues, Survival Rates, and Mean Arterial Pressure of Mice With Lipopolysaccharide-Induced Shock

**FIGURE 1:**
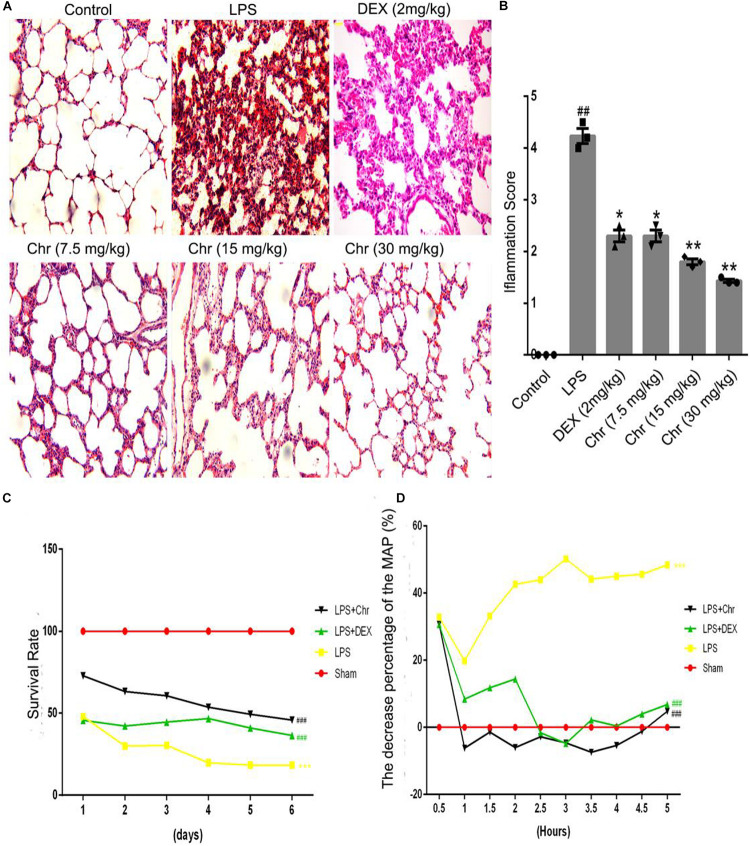
Effects of Chr on the pathological changes, survival rate, and MAP of ALI mice (*n* = 12). **(A,B)** Pathological effects of Chr (7.5, 15, 30 mg/kg) in lung tissues (×200). **(C)** Survival rate of different groups. **(D)** MAP was recorded over 5 h at 30 min intervals. ^#^*P* < 0.01, ^##^*P* < 0.01, ^###^*P* < 0.001 vs. control group; *P < 0.05, **P < 0.01, ***P < 0.001 vs. LPS group.

We used hematoxylin–eosin staining to reveal the pathological changes on pulmonary tissue. Compared with the control group, the LPS group presented distinct neutrophil sequestration and infiltration in the lung tissue. The Chr- and DEX-treated groups showed varying degrees of protective effects in the LPS-induced mouse model (Figures 1A,B).

As an essential indicator of the therapeutic outcome of Chr, the survival rate of mice was measured (Figure 1C). Administration of 15 mg/kg LPS led to 70% mice mortality (*P* < 0.001). Chr treatment (Chr 30 mg/kg) protected mice from LPS-induced lethality, as shown by the higher survival rates; in addition, survival rates were better than those of the DEX group. Next, we evaluated the impact of Chr on MAP in our mouse model. LPS administration lowered the MAP by > 30% (*P* < 0.01). In contrast, Chr significantly restored the reduction in MAP in ALI mice, more strongly than with DEX treatment (Figure 1D).

### Effects of Chrysophanol on Wet-to-Dry, Myeloperoxidase in Lung Tissues, Malondialdehyde, and Superoxide Dismutase Activities in Bronchoalveolar Lavage Fluid

To estimate the degree of pulmonary edema, the W/D weight ratio, MDA, and SOD levels in BALF were calculated.

**FIGURE 2:**
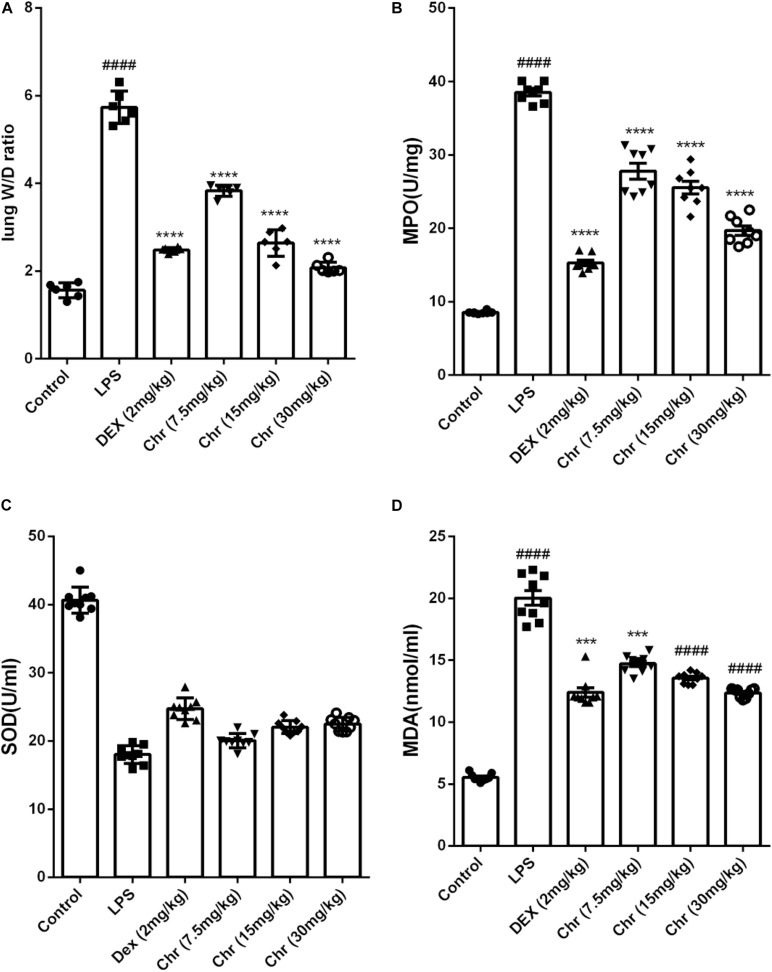
Effects of Chr on **(A)** wet-to-dry weight ratio, **(B)** MPO, **(C)** SOD, and **(D)** MDA activities. Data are expressed as mean ± *SD*. ^#^*P* < 0.05, ^##^*P* < 0.01, ^####^*P* < 0.0001 compared with control group, *P < 0.05, **P < 0.01, ***P < 0.001, ****P < 0.0001 compared with LPS.

Chr notably decreased the LPS-induced lung W/D weight ratio (Figure 2A). LPS sharply increased the production of MPO and MDA, whereas Chr treatment (7.5, 10, and 20 mg/kg) remarkably decreased MPO and MDA levels in the BALF (Figures 2B,D). The SOD index was downregulated in the LPS-induced sepsis model group, and Chr (7.5, 10, and 20 mg/kg) treatment led to a significant increase in SOD activity in BALF (Figure 2C).

### Chrysophanol Inhibited High Mobility Group Protein 1/Nuclear Transcription Factor-Kappa B p65 Signaling Pathway Activity in the Acute Lung Injury Model

**FIGURE 3:**
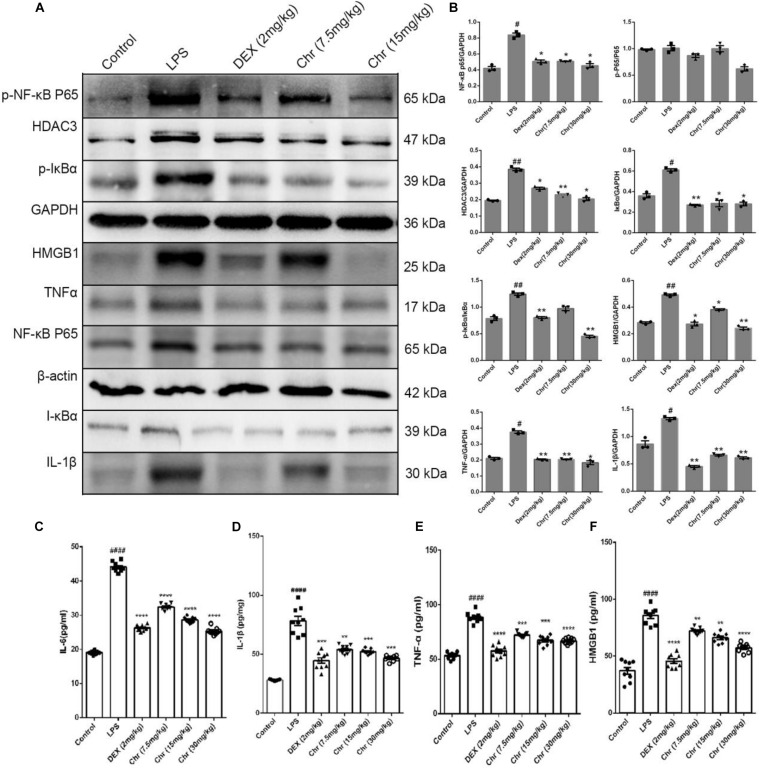
Effects of Chr on HMGB1/NF-κB axis in mice. **(A,B)** Protein content of inflammation factors was analyzed by Western blotting. **(C–F)** Influence of Chr on TNF-α, IL-6, IL-1β, and HMGB1 concentration in BALF as measured by ELISA. Data are expressed as mean ± *SD*. ^#^*P* < 0.05, ^##^*P* < 0.01, ^####^*P* < 0.0001 vs. control; *P < 0.05, **P < 0.01, ***P < 0.001, ****P < 0.0001 vs. LPS.

As shown in Figures 3A,B, we explored the inflammatory response in murine lung tissue. We detected NF-κB pathway-related proteins using WB analysis. The expression of NF-κBp65, p-NF-κB p65, IκBα, p-IκBα, HMGB1, TNF-α, and IL-1β increased in the LPS-treated group. As anticipated, Chr (7.5 and 15 mg/kg) or DEX treatment significantly inhibited the expression of these proteins. The results indicated that Chr possessed the anti-inflammatory activity in treating LPS-induced ALI through the HMGB1/NF-κB pathway. Moreover, we also found that Chr significantly downregulated LPS-induced HDAC3 protein levels. The ELISA result confirmed that the LPS group presented significantly enhanced TNF-α, IL-6, IL-1β, and HMGB1 release in BALF; however, their concentrations were decreased after Chr treatment (Figures 3C–F). These data indicated that Chr could sharply inhibit the levels of inflammatory factors in the ALI mouse model.

### Effects of Chrysophanol on the Viability of RAW264.7 Cells

**FIGURE 4:**
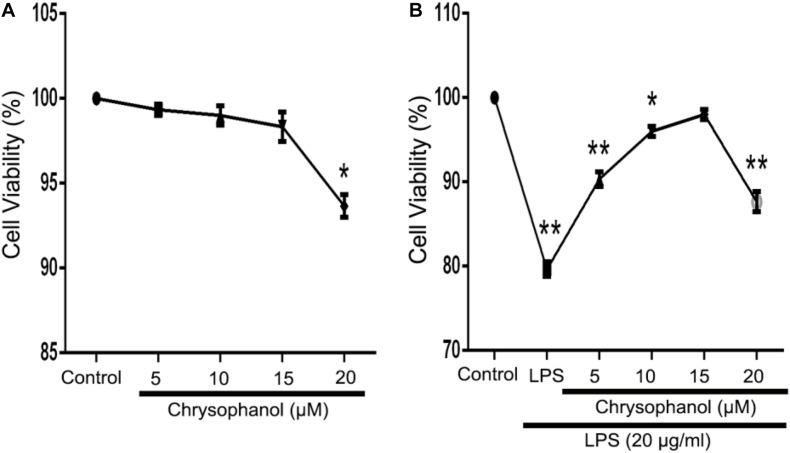
Effects of Chr on the viability of RAW264.7 cells. MTT assay was used to evaluate effects of **(A)** Chr in RAW264.7 cells and in **(B)** LPS-stimulated model pretreated with Chr with increasing concentration of Chr. *P < 0.05, **P < 0.01 vs. control.

We used the MTT assay to investigate the effects of Chr at different concentrations (5, 10, 15, or 20 μM) on RAW264.7 cells viability in the LPS-induced *in vitro* cell model. There was no obvious cytolethality induced by Chr on cells (Figure 4A), except for the 20 μM Chr-treatment group. Instead, the viability of cells stimulated by LPS was 80.0 ± 1.0% of the control group, and treatment with Chr (5, 10, and 15 μM) protected cells against LPS-induced cellular injury, increasing cell activity up to 90.67 ± 1.15%, 96.0 ± 1.73%, and 98.0 ± 1.0%, respectively (Figure 4B). Therefore, Chr (5, 10, and 15 μM) were decided to use for the next experiment.

### Chrysophanol Reduced Lipopolysaccharide-Induced High Mobility Group Protein 1 Expression, Nucleocytoplasmic Translocation, and Acetylation in Macrophages

**FIGURE 5:**
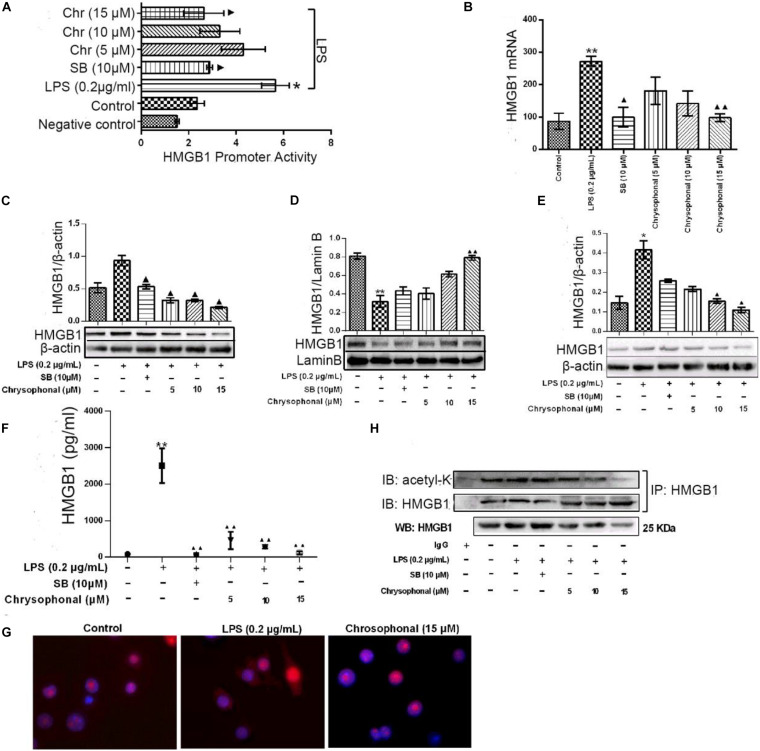
Chr reversed LPS-induced HMGB1 release and inhibited HMGB1 acetylation. Cells were treated with LPS (0.2 μg/ml) for 30 min before being exposed to Chr (5, 10, and 15 μM) for 24 h. **(A)** HMGB1 promoter activity was determined. Mean value of the relative luciferase activity is shown. **(B)** Inhibition of HMGB1 mRNA expression by Chr in RAW264.7 cells. **(C)** Protein expression of HMGB1. **(D)** Protein expression of the nuclear HMGB1. **(E)** Protein expression of cytosolic HMGB1. **(F)** HMGB1 concentration as determined by ELISA. **(G)** Localization of HMGB1 was visualized in RAW264.7 cells by confocal microscopy. **(H)** Western blotting was performed to determine acetylated HMGB1 levels. Data are presented as means ± *SD* for three independent experiments. *P < 0.05, **P < 0.01 vs. control; ^▲^*P* < 0.05, ^▲^^▲^*P* < 0.01 vs. LPS.

Our previous study found Chr could attenuate inflammatory factor levels in LPS-induced macrophages. To investigate the potential role of Chr, we examined the effects of Chr on HMGB1 *in vitro*. We evaluated HMGB1 promoter activity and mRNA levels. Treatment with Chr (5, 10, and 15 μM) and the HMGB1 inhibitor SB (10 μM) significantly inhibited LPS-induced HMGB1 promoter activity (Figure 5A) and mRNA expression (Figure 5B), compared with the LPS-treatment group. We also evaluated the influence of Chr on total, cytoplasmic, and nuclear HMGB1 protein levels by WB. As shown in Figures 5C–E, total and cytoplasmic HMGB1 levels increased, whereas the nuclear levels decreased after LPS stimulation. However, all these effects were reversed by exposure to Chr.

We then examined the levels of HMGB1 in the supernatant by ELISA assay. The levels of HMGB sharply increased after LPS exposure; however, this increase was inhibited in the presence of 10 μM SB (Figure 5F). Furthermore, Chr nearly completely abolished LPS-triggered intracellular HMGB1 production, similar to that in the SB-treated group (Figure 5F). Our results indicated the important inhibitory activity by Chr (5, 10, and 15 μM) on LPS-induced HMGB1 release in a dose-dependent fashion.

Immunofluorescent assay results also supported our conclusion. Nuclear–cytoplasmic translocation of HMGB1 was induced by LPS (Figure 5G, middle). Simultaneous treatment with Chr reduced cytoplasmic HMGB1 and enhanced nuclear localization of HMGB1 (Figure 5G, right), similar to unstimulated cells (Figure 5G, left), which provided morphological evidence to support our results.

To explore whether the HMGB1 translocation is related to its deacetylation, we analyzed the content of acetylated HMGB1 by IP (Figure 5H). When LPS-induced RAW264.7 cells to release HMGB1, we found acetylated HMGB1 levels were enhanced. Conversely, HMGB1 acetylation was markedly decreased after Chr treatment, indicating that Chr interfered with the acetylation of HMGB1 (Figure 5H).

### Effects of Chrysophanol on Nuclear Transcription Factor-Kappa B Pathway After Histone Deacetylase 3 Knockdown

**FIGURE 6:**
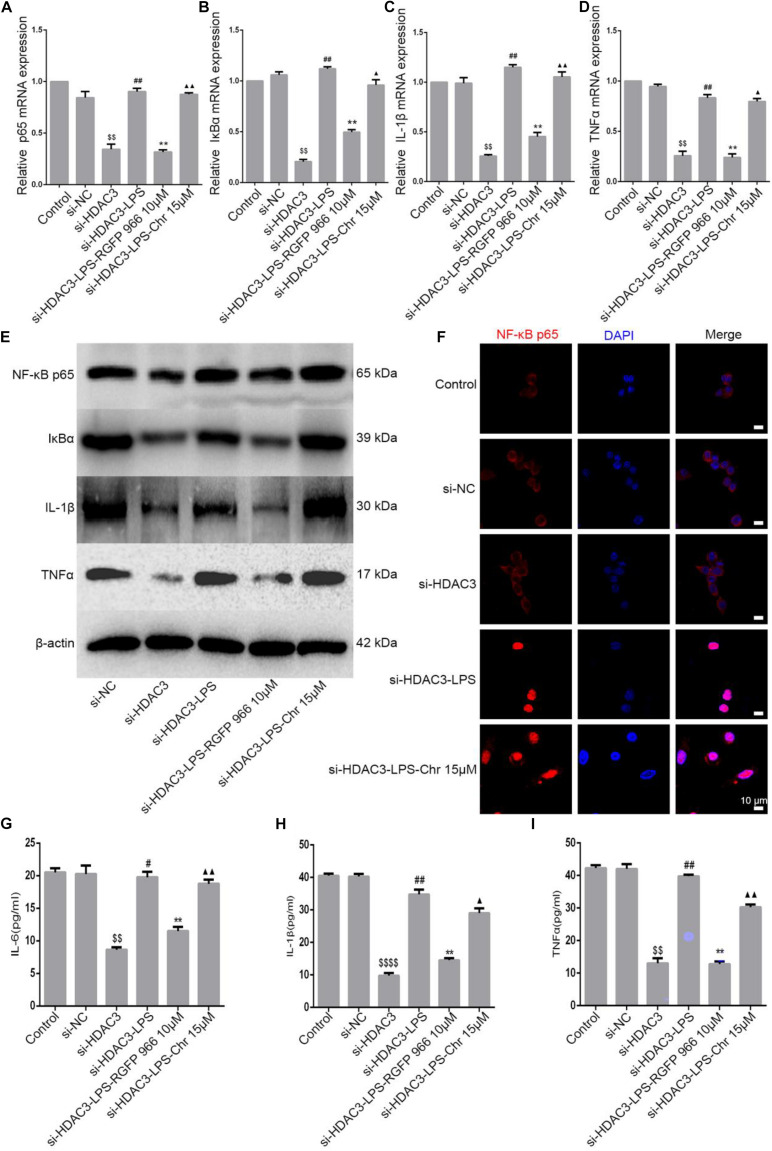
Effect of Chr on NF-κB pathway after HDAC3 knockdown. siHDAC3 was transfected into RAW264.7 cells. **(A–D)** Expression of mRNA levels of NF-κB signaling pathway. **(E)** Protein expression of components of NF-κB signal pathway. **(F)** Immunofluorescence staining results. **(G–I)** Concentrations of NF-κB downstream factors as determined by ELISA. Data shown are mean ± *SD*. ^$^*P* < 0.05, ^$$^*P* < 0.01, ^$$$$^*P* < 0.0001 vs. control group; ^#^*P* < 0.05, ^##^*P* < 0.01 vs. siHDAC3 group; *P < 0.05, **P < 0.01, vs. siHDAC3-LPS group; ^▲^*P* < 0.05, ^▲^^▲^*P* < 0.01, vs. RGFP966 group.

Our previous study demonstrated that Chr significantly reduced LPS-activated NF-κB promoter and the expression of mRNA, protein, and phosphorylation of components of the NF-κB pathway (Wen et al., 2018). Because Chr significantly downregulated HDAC3 protein expression and inhibited NF-κB pathway activation in the sepsis shock mice model, we then performed *in vitro* model experiments to clarify the potential mechanisms involved. To specifically knockdown HDAC3 levels, we used targeted siRNA inhibition. After LPS induction, Chr (15 μM) and HDAC3 antagonist RGFP966 (10 μM) were added to cells for 24 h. Then, qPCR and WB were performed. As shown in Figures 6A–E, mRNA and protein expression (NF-kB p65, IκBα, IL-1β, and TNFα) in the control group and siNC group were unchanged. Instead, after siHDAC3 transfection, the mRNA and protein levels of NF-kB p65, IκBα, IL-1β, and TNFα were more remarkably downregulated than the control group (*P* < 0.01). After transfected HDAC3 siRNA, in contrast with the siHDAC3-treated group, the NF-kB p65 pathway activity was enhanced by LPS (*P* < 0.01), exposure to RGFP966 significantly inhibited LPS-enhanced NF-kB p65 pathway activation (*P* < 0.05). In contrast, we observed that pathway inhibition by Chr treatment was reversed after HDAC3 gene knockdown (Figure 6).

Next, immunofluorescence analysis was performed. NF-κB p65 nuclear localization signals were increased in the siHDAC3-LPS group, consistent with the expression of inflammation genes, HDAC3 knockdown almost entirely abrogated the inhibitory capacity of Chr-modulated NF-κB p65 nuclear translocation (Figure 6F).

In brief, the Chr inhibitory effect on NF-κB p65 expression and on downstream proinflammatory gene expression was notably reversed in cells transfected with siRNA targeting HDAC3.

### Chrysophanol Reduced Lipopolysaccharide-Induced High Mobility Group Protein 1 Acetylation and Translocation Were Reversed After Knockdown of Histone Deacetylase 3 Expression by Small Interfering RNA

**FIGURE 7:**
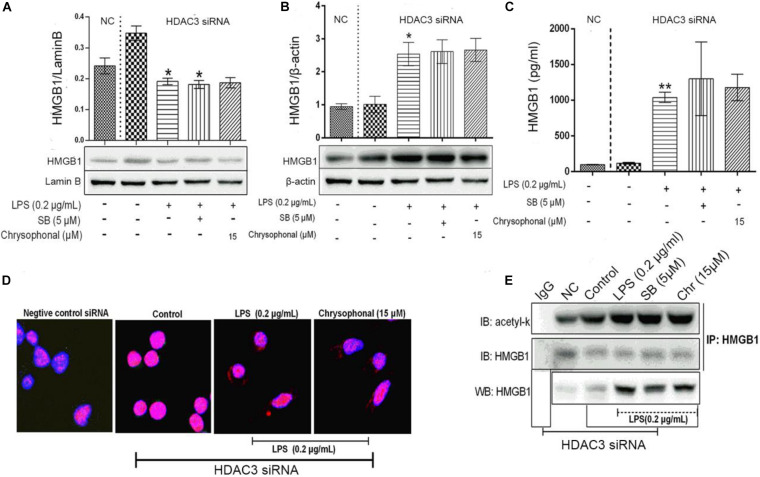
Knockdown of HDAC3 expression promotes nuclear HMGB1 translocation to the cytoplasm, enhances HMGB1 release, and reverses Chr effects. **(A)** Protein expression of nuclear HMGB1. **(B)** Protein expression of cytosol HMGB1. **(C)** HMGB1 concentration. **(D)** Localization of HMGB1was visualized in RAW264.7 cells by confocal microscopy. Red, HMGB1; blue, nuclei. **(E)** Western blotting was performed to determine acetylated HMGB1 levels. Data are analyzed as means ± *SD* for three independent experiments. *P < 0.05, **P < 0.01 *vs*. control group.

As a part of the HDAC protein family, HDAC3 has been reported to play a key role in blocking HMGB1 secretion (Bonaldi et al., 2003). To further investigate the influence of Chr on HDAC3 activity and levels of acetylated HMGB1 and cytoplasmic to nuclear translocation, we transfected HDAC3 siRNA and NC-siRNA into RAW264.7; in addition, the HMGB1 inhibitor SB was used as a positive control. The distribution of HMGB1 was analyzed to determine the nucleocytoplasmic localization using fluorescence signals, HMGB1production was analyzed through ELISA, and IP was used to detect the HMGB1 acetylation level. As shown in Figures 7A,B, LPS induced translocation of HMGB1 protein from the nucleus to the cytoplasm; meanwhile, the inhibitory effects on HMGB1 translocation by Chr treatment were reversed in HDAC3 siRNA-transfected cells. In addition, ELISA results showed that HDAC3 siRNA eliminated the inhibitory effects of Chr on HMGB1 production in the cell culture supernatants (Figure 7C).

Immunofluorescent staining for HMGB1 recapitulated the results of WB and ELISA. In the HDAC3 siRNA control group and siRNA NC group, HMGB1 staining was only positive in the nucleus, whereas HMGB1 fluorescence signals were present in both the nuclear and cytoplasmic compartments in both the LPS- and the Chr-treated groups (Figure 7D). As shown in Figure 7E, after siRNA transfection, Chr treatment did not reduce LPS-induced HMGB1 acetylation.

Collectively, these results demonstrated that Chr reversed LPS-triggered HMGB1 acetylation and nucleocytoplasmic translocation by enhancing HDAC3 expression.

### Chrysophanol Enhances Formation of the HDAC3:HMGB1:NF-κB p65 Complex

**FIGURE 8:**
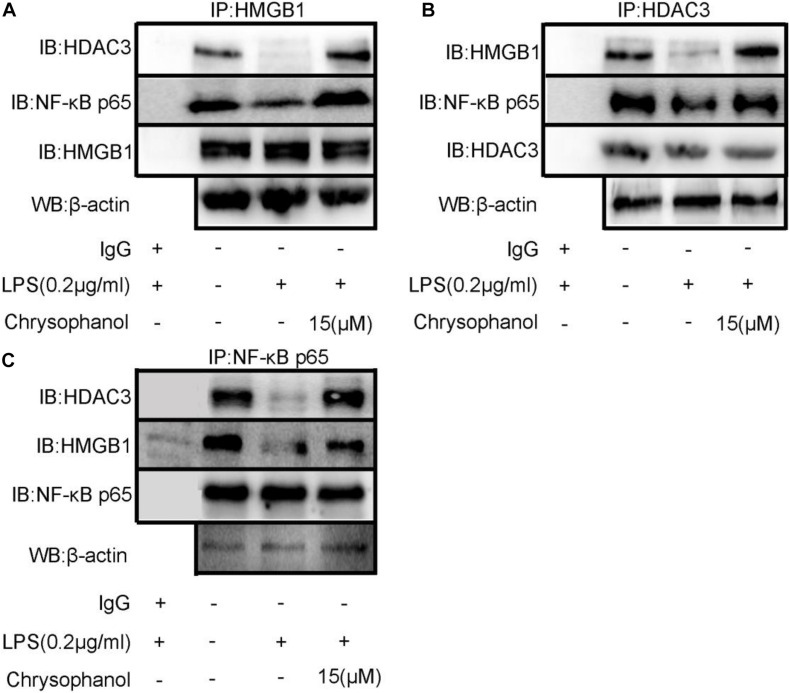
Chr enhances interaction between HDAC3, HMGB1, and NF-κB p65. Immunoprecipitation of total and **(A)** anti- HMGB1-, **(B)** anti- HDAC3-, or **(C)** anti-NF-κB p65-antibody-treated lysates, respectively, subjected to WB.

Protein–protein interactions have an important role in multiple diseases (Gao et al., 2006). We speculated that HDAC3 deacetylates HMGB1 and regulates the NF-κB p65-mediated inflammatory signaling pathway by enhancing the interaction between HDAC3:HMGB1:NF-κB p65. As shown in Figure 8, LPS-induced RAW264.7 cell extracts were immunoprecipitated with HMGB1, HDAC3, and NF-κB p65 antibodies, respectively, and were subjected to WB using HDAC3, NF-κB p65, and HMGB1 antibodies. The results indicated a positive interaction between these proteins. After stimulation with LPS, the amount of HDAC3 immunoprecipitated with anti-HMGB1, NF-κB p65 antibodies were decreased. By comparison, the interaction of HMGB1:NF-κBp65 and HMGB1:HDAC3 were dramatically enhanced through treatment with 15 μM Chr (Figure 7A). Moreover, similar results were observed when the cell extract was immunoprecipitated with the HDAC3 or NF-κB p65 antibody and followed by immunoblotting with HMGB1, NF-κB p65, or HDAC3, HMGB1 antibodies (Figures 8B,C).

Results verifying the relationship of the three proteins indicated that LPS induced HMGB1, NF-κB p65, and HDAC3 complex dissociation, whereas Chr enhanced the interaction between HMGB1, NF-κB p65, and HDAC3, which changes reflected our intended meaning.

### Histone Deacetylase 3 Knockdown Suppressed Chrysophanol-Mediated Enhancement of HDAC3:HMGB1:NF-κB p65 Complex Formation

**FIGURE 9:**
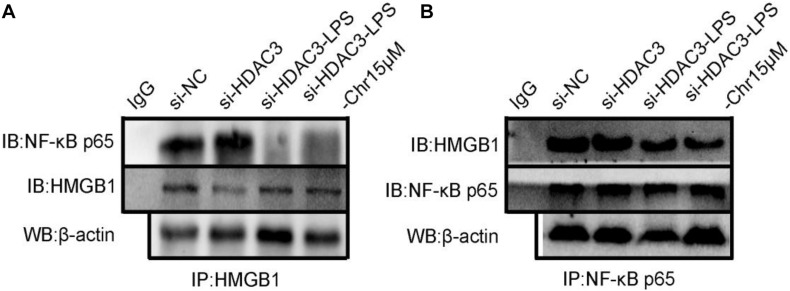
**(A)** Co-immunoprecipitation was performed using protein lysate from cells to observe relationship between HMGB1/NF-κB p65 complex formation in HDAC3 knockdown condition with specific siRNA and after LPS activation and Chr treatment. **(B)** Co-immunoprecipitation was performed to determine the interactions between p65-HMGB1.

Then, we used siRNA to knockdown HDAC3 expression *in vitro*. HDAC3 was decreased after transfection into RAW264.7 cells with siRNA for 48 h. Co-immunoprecipitation assays were carried out to test the interaction between the HDAC3:HMGB1:NF-κB p65 protein complex after HDAC3 knockdown by HMGB1 and NF-κB p65 antibodies, respectively. We found that under the NC or HDAC3-siRNA conditions, HMGB1 protein was detected in the IP product using the p65 antibody, and conversely, p65 protein was detected in the IP product using the HMGB1 antibody (Figures 9A,B). After LPS stimulation, the coexisting protein–protein interactions between HMGB1 and p65 were inhibited, which was observed in the siHDAC3-LPS group. Moreover, we observed from the IP product that HDAC3 knockdown potently abolished the Chr-augmented p65:HMGB1 proteins complex formation, indicating the regulation role of HDAC3 in Chr-mediated enhancement of HDAC3:HMGB1:NF-κB p65 complex formation.

## Discussion

Sepsis is a life-threatening pathological condition characterized by a dysregulated inflammatory response, a disordered blood coagulation cascade, and multiple organ dysfunctions (Falagas and Kopterides, 2006; Jean-Baptiste, 2007; Prescott et al., 2016). The effective treatment of sepsis involves the inhibition of the expression of proinflammatory mediators. In our study, we examined the underlying mechanisms and protective effects of Chr in LPS-induced ALI in *in vitro* cell lines and in an *in vitro* LPS-induced murine model of ALI. Our findings explicitly demonstrated that Chr could significantly downregulate the HMGB1/NF-κB axis and inhibit the inflammatory response via modulation of HDAC3 expression. Finally, Chr treatment markedly enhanced protein–protein interactions and relieving symptoms of ALI.

The overproduction of inflammatory mediators has been associated with the pathogenesis of ALI; thus, the identification of effective measures to control lung injury is important. The therapeutic activity of DEX is attributed to its anti-inflammatory (Tolaj et al., 2017), immunosuppressive (Wang et al., 2013), anti-endotoxic (Zhang et al., 2017), and anti-shock (Yang et al., 2010) activity. Recent studies have determined that DEX possesses the capacity to activate the remission of organ injury and to overcome the infection, although the limitation of DEX treatment is that it is not recommended for long-term use, as it causes numerous adverse effects (Wang et al., 2013; Sun et al., 2017). Thus, there is an urgent need to identify novel effective treatments for ALI having fewer secondary adverse effects.

LPS induces the same disease characteristics as ALI and thus has been applied as the best model for ALI. We studied the protective effects of Chr in the ALI mouse model. The results showed that Chr sharply reduced MAP, the lung W/D weight ratio, lung MPO activity, MDA content in LPS-stimulated mice, and the levels of several inflammatory mediators in the BALF. In addition, Chr also increased SOD levels and the survival rate and, thus, effectively relieved ALI.

The level of lysosomal-specific MPO is an indicator of the active condition of macrophages (Yang et al., 2016), which contribute to inducing the oxidative stress reaction and causing organ or tissue damage (Jiang et al., 2016). Researchers have determined that the lipid peroxidation reaction induced by MDA (Diao et al., 2016) can lead to substantial cytotoxicity. SOD has been reported to be the major enzymatic antioxidative enzyme able to detoxify and eliminate free radicals (Wang et al., 2016). Our study suggested that Chr treatment could enhance SOD levels in contrast to the changes observed in the LPS group.

We then performed WB and ELISA to identify mechanisms involved in Chr protection against LPS-induced ALI. ALI is well known to develop from the increased exposure to cytokines released during the inflammation process. Our results indicated that the activation of LPS led to a significant increase in the activation of the NF-κB p65 pathway, as detected in the BALF, whereas Chr treatment disrupted the LPS-induced pro-inflammatory effects. Bacterial LPS triggering activation of the NF-κB signaling pathway has been considered to be a major component of ALI. LPS interacts with TLRs. In addition, as a late-onset risk factor, released HMGB1 induces the inflammatory cascade and accelerates the synthesis of inflammatory cytokines by binding to NF-κB receptors, which triggers NF-κB kinase phosphorylation, and signals that converge on the IkB/NF-kB, allowing the disruption of the complex, and shifting of NF-κB to the nucleus, leading to the release of large quantities of lethal factors (Jiang et al., 2005). Moreover, the results suggested that Chr treatment decreased LPS-enhanced expression of HDAC3, which is considered a transcriptional co-repressor of NF-κB p65. The HDAC3 inhibitor effectively inhibited NF-κB p65 transcriptional activity, likely by binding to the co-repressor transcription complex PPARγ (Jennewein et al., 2008).

In line with our *in vivo* study, we assumed that Chr could also inhibit activated cells and intrapulmonary lethal cytokines and relieve ALI through the HMGB1/NF-κB pathway via the HDAC3 signaling. The *in vitro* study was designed to define better the underlying molecular mechanisms involving Chr activity on the HMGB1/NF-κB signaling pathway. First, we found that Chr suppressed LPS-induced HMGB1 acetylation, which was in line with the reduction in HMGB1 nuclear translocation and extracellular HMGB1 production, suggesting a mechanism in which HDAC3 deacetylates HMGB1 and leads to the inhibition of HMGB1 release. Our previous study confirmed that Chr treatment inhibited the NF-κB p65 signaling pathway via inhibition of sIκB phosphorylation and NF-κB p65 relocation triggered by LPS-activated RAW264.7 cells (Wen et al., 2018). To provide a more in-depth exploration of how and whether Chr-regulated HMGB1 suppression influenced the HDAC3-mediated inactivation of NF-kB p65, we used synthetic siRNA targeting HDAC3. We found that the inhibitory effect of Chr was essentially lost in cells where the expression of HDAC3 was silenced, suggesting an important role for HDAC3 in the effects induced by Chr.

**FIGURE 10:**
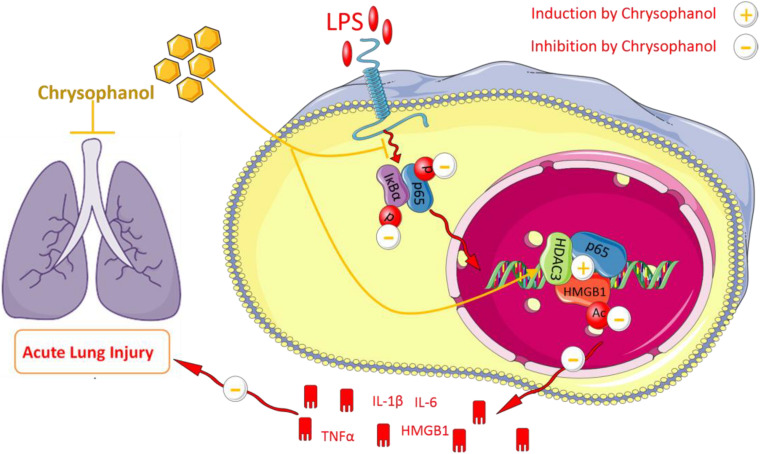
Schematic diagram of Chrysophanol-mediated suppression of the inflammatory response.

Next, we explored the mechanisms involved in Chr-regulation of the p65:HDAC3:HMGB1 complex. Co-immunoprecipitation findings indicated that the members of the p65:HDAC3:HMGB1 complex were tightly bound to each other in the quiescent state. Then, we confirmed that after LPS activation, the binding between the components of the complex was disrupted, which might be a consequence of protein degradation. Furthermore, we showed that Chr treatment before LPS stimulation led to the stronger interaction between the complex components. When HDAC3 was knocked down, the enhancement effect of Chr was sharply abolished, suggesting an essential role of HDAC3 in the effects of Chr (Figure 10). In brief, our research showed that Chr possesses an intense anti-inflammatory effect by regulating HMGB1/NF-κB signaling through HDAC3.

Our study has some limitations. In our experiments, the potential mechanism of Chr via macrophages *in vivo* was not investigated. Thus, to better understand and characterize LPS-induced ALI, influence functions of other cell types (e.g., pulmonary macrophages and epithelial cells) are required to assess the clinical benefits of Chr further.

## Conclusion

In conclusion, the present study showed that Chr exerts an intense anti-inflammatory effect by regulating HMGB1/NF-κB signaling through HDAC3 expression.

## Data Availability Statement

The datasets presented in this study can be found in online repositories. The names of the repository/repositories and accession number(s) can be found in the article/Supplementary Material.

## Ethics Statement

The animal study was reviewed and approved by the Animal Ethics Committee of Guangzhou University of Chinese Medicine.

## Author Contributions

QW and NL conceived and designed the experiments. QW performed the experiments, analyzed the data, prepared manuscript, and wrote the manuscript. NL contributed to the reagents, materials, and analysis tools. All authors read and approved the manuscript.

## Conflict of Interest

The authors declare that the research was conducted in the absence of any commercial or financial relationships that could be construed as a potential conflict of interest.
