# Supplementary material for: Chrysophanol Exerts Anti-inflammatory Activity by Targeting Histone Deacetylase 3 Through the High Mobility Group Protein 1-Nuclear Transcription Factor-Kappa B Signaling Pathway in vivo and in vitro
Source: Front Bioeng Biotechnol. 2021 Jan 25;8:623866. doi: 10.3389/fbioe.2020.623866 (PMC7868569; doi:10.3389/fbioe.2020.623866)
Supplement: Supplementary file 1 [file Data_Sheet_1.docx]

**Effect of Chr on the BrdU of RAW264.7 cells**

**Materials and methods**

**BrdU Incorporation**

A total of 1 × 10^4^ cells per well were seeded in a 12-well plate and allowed to adhere on coated slides. Cells were treated with Chr for the time indicated. After Chr exposure, BrdU was added. Cells were fixed and stained with DAPI and anti-BrdU antibodies. Cells were photographed using a fluorescence microscope equipped with a 40× objective. Seven sets of images were obtained from different areas, the total amount of cells (DAPI) and the number of BrdU-positive cells was calculated.

**Result**

In line with MTT assay, Chr had no inhibition effect on the proportion of cells in the cell cycle (Fig. S1).


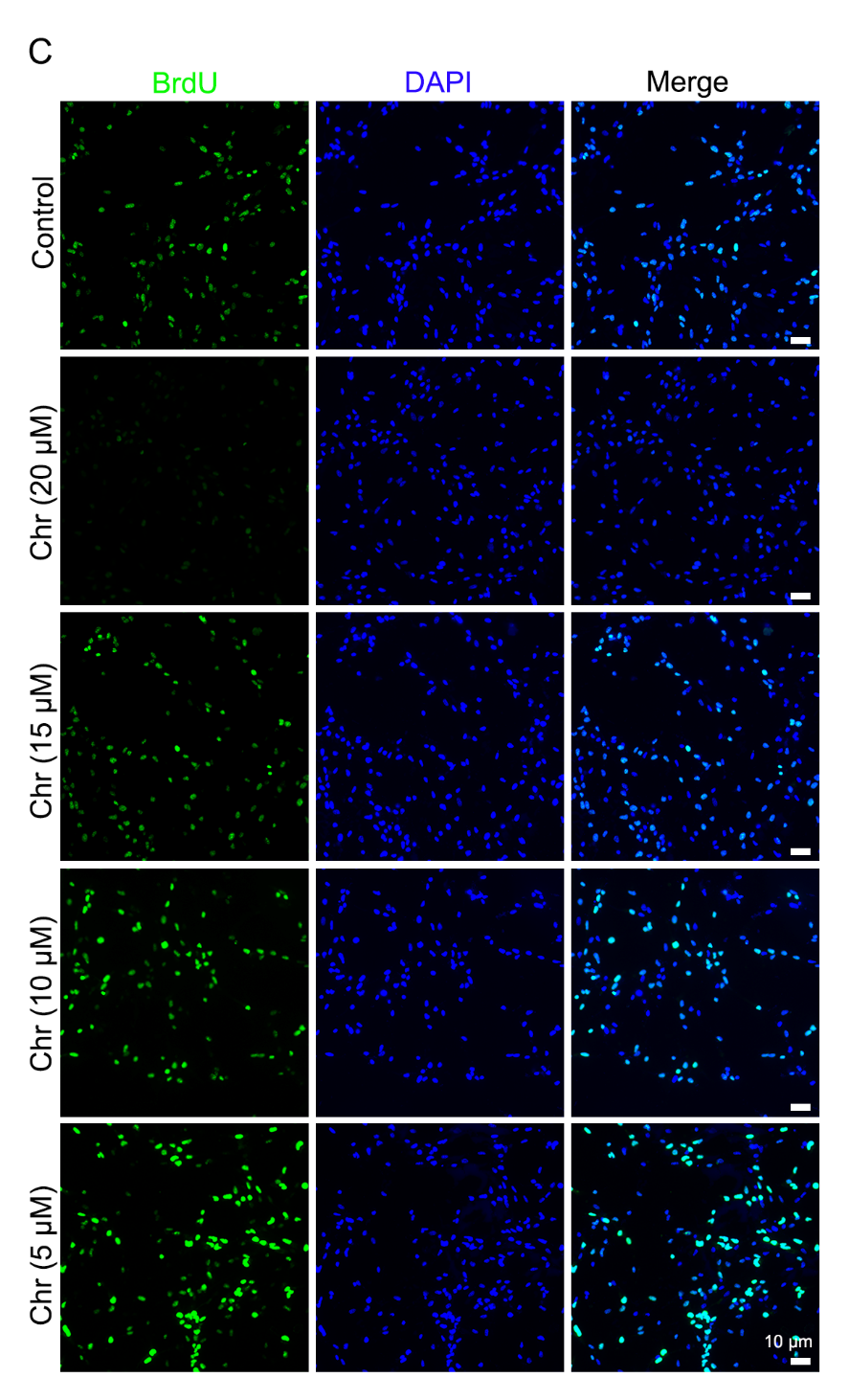


Figure. Supplemental.1 Cells were given BrdU after 24 h treated with Chr then was performed using immunofluorescence staining.
